# Subcellular distribution of the V-ATPase complex in plant cells, and *in vivo *localisation of the 100 kDa subunit VHA-a within the complex

**DOI:** 10.1186/1471-2121-5-29

**Published:** 2004-08-13

**Authors:** Christoph Kluge, Thorsten Seidel, Susanne Bolte, Shanti S Sharma, Miriam Hanitzsch, Beatrice Satiat-Jeunemaitre, Joachim Roß, Markus Sauer, Dortje Golldack, Karl-Josef Dietz

**Affiliations:** 1 Biochemistry and Physiology of Plants – W5, University of Bielefeld, Bielefeld, 33501, Germany; 2 CNRS, UPR 2355, Institut des Sciences du Végétale, Avenue de la terrasse, Gif Sur Yvette, 91198, France; 3 Department of Biosciences, H. P. University, Shimla, 171 005, India; 4 Applied Laser Physics and Laser Spectroscopy – D3, University of Bielefeld, Bielefeld, 33501, Germany

## Abstract

**Background:**

Vacuolar H^+^-ATPases are large protein complexes of more than 700 kDa that acidify endomembrane compartments and are part of the secretory system of eukaryotic cells. They are built from 14 different (VHA)-subunits. The paper addresses the question of sub-cellular localisation and subunit composition of plant V-ATPase *in vivo *and *in vitro *mainly by using colocalization and fluorescence resonance energy transfer techniques (FRET). Focus is placed on the examination and function of the 95 kDa membrane spanning subunit VHA-a. Showing similarities to the already described Vph1 and Stv1 vacuolar ATPase subunits from yeast, VHA-a revealed a bipartite structure with (i) a less conserved cytoplasmically orientated N-terminus and (ii) a membrane-spanning C-terminus with a higher extent of conservation including all amino acids shown to be essential for proton translocation in the yeast. On the basis of sequence data VHA-a appears to be an essential structural and functional element of V-ATPase, although previously a sole function in assembly has been proposed.

**Results:**

To elucidate the presence and function of VHA-a in the plant complex, three approaches were undertaken: (i) co-immunoprecipitation with antibodies directed to epitopes in the N- and C-terminal part of VHA-a, respectively, (ii) immunocytochemistry approach including co-localisation studies with known plant endomembrane markers, and (iii) *in vivo*-FRET between subunits fused to variants of green fluorescence protein (CFP, YFP) in transfected cells.

**Conclusions:**

All three sets of results show that V-ATPase contains VHA-a protein that interacts in a specific manner with other subunits. The genomes of plants encode three genes of the 95 kDa subunit (VHA-a) of the vacuolar type H^+^-ATPase. Immuno-localisation of VHA-a shows that the recognized subunit is exclusively located on the endoplasmic reticulum. This result is in agreement with the hypothesis that the different isoforms of VHA-a may localize on distinct endomembrane compartments, as it was shown for its yeast counterpart Vph1.

## Background

Vacuolar H^+^-ATPases are large multi-heteromeric protein complexes located at endomembranes of all eukaryotic cells. Plant V-ATPase has been identified at the vacuolar and various other endomembranes, and also at the plasma membrane [1-3]. The total molecular mass of V-ATPase is estimated to surpass 700 kDa. V-ATPase pumps protons into membrane-surrounded intracellular compartments at the expense of hydrolysis energy of ATP [4]. V-ATPases share a common structure composed of a ball-like head, a membrane-intrinsic part and connecting stalks similar to ATP-producing F-ATP synthases. Biochemical examinations of the subunit composition revealed that V-ATPases are built from up to 14 subunits. The protein subunits are denominated VHA-A through H for soluble components protruding into the cytoplasm (V_1_-part), and VHA-a, c, c', c", d and e for membrane-associated subunits (V_0_-part) [5]. In plants, the molecular characterisation of VHA-subunits is only beginning to include also the genes that were identified recently [6,7]. Following the cloning of cDNA sequences coding for VHA-A, -B, -c, and -E until 1995, VHA-D, -F, -C and -G were cloned from *A. thaliana*, oat and barley [5,7]. However, the first complete set of subunit sequences only became available with the sequencing of the genome of *A. thaliana *[9]. The second set was cloned from the halotolerant, facultative CAM plant *Mesembryanthemum crystallinum *[10], and a third set is now available from rice. A comparative analysis of the genes/ESTs revealed that *Arabidopsis *and *Mesembryanthemum *express a similar set of subunits [7]. A detailed analysis of the sequences from both species showed that all examined subunits share the same properties in binding of ATP and conducting protons through the proteolipid ring [10]. An important question concerns the composition of the plant V_0_-sector since its subunit composition is not resolved yet. VHA-a is the subunit with the highest molecular mass within the V-ATPase and reveals a bipartite structure. As first shown in yeast, VHA-a consists of a 50 kDa hydrophilic N-terminal domain and a hydrophobic, membrane-spanning C-terminus [11-13]. Interestingly, Li and Sze [14] could not observe the VHA-a subunit in functional V-ATPase of oat. Therefore, they suggested a role of VHA-a in assembly of plant V-ATPase. The conclusion is in contrast to results in yeast where site directed mutagenesis had allowed to identify amino acids involved in the mechanism of proton translocation across the membrane. Mutant yeast cells devoid of VHA-a or complemented with modified VHA-a variants exhibited the conditional phenotype of V-ATPase deficiency [15]. Another novel subunit in plants that was only addressed recently is VHA-H. VMA13p being the yeast orthologue of VHA-H was cloned and represents the only crystallized subunit of V-ATPase at present [16]. VHA-H is considered to activate and regulate V-ATPase by functionally coupling ATP hydrolysis to proton flow through the V_o_-domain [17].

The aim of the study was to improve the understanding of V-ATPase distribution in plant cells with emphasis on the localization of VHA-a and VHA-H in the V-ATPase structure. Three specific questions were answered using different methodology: (i) Is VHA-a part of the V-ATPase structure? (ii) Where are different subunits localized within plant cells? (iii) Is FRET a suitable method to investigate subunit interaction within the complex, for example between VHA-a and VHA-c, and VHA-H and VHA-B.

## Results

### Primary structure of VHA-a

**Figure 1:**
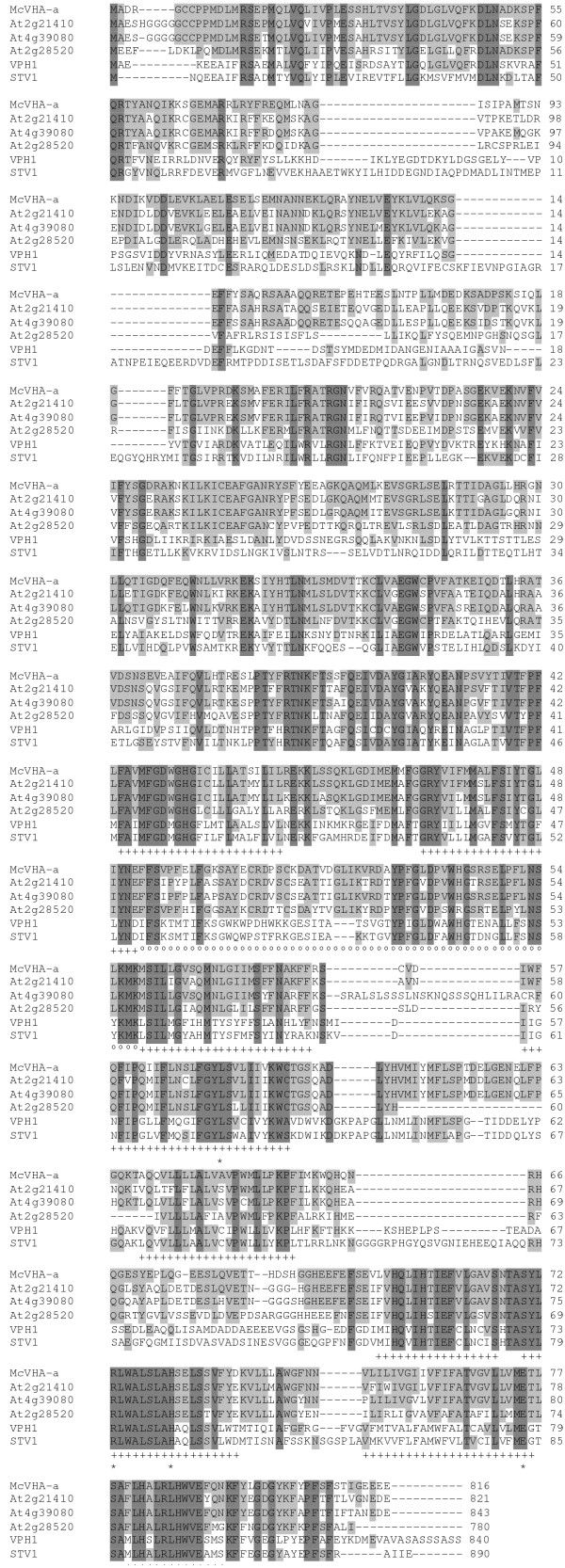
**Amino acid sequence alignment of plant and yeast VHA-a. **Comparison of amino acid sequences deduced from the three coding regions detected in the *A. thaliana *genome, the sequence of *M. crystallinum *and the two yeast gene products Vph1 and Stv1 using ClustalW-algorithm. Identical amino acids are marked in dark grey, those similar with *M. crystallinum *in light grey. The programs JPRED (EMBL, Hinxton) and THMM (EBS, Denmark) were used to predict transmembrane domains (labelled with +). Amino acids identified as essential for H^+^-pumping in yeast are marked with *.

From the database dbEST (NCBI, USA) a cDNA fragment from *M. crystallinum *was identified (AI822404) with similarity to known genes coding for VHA-a in yeast. Using RACE-PCR a full length cDNA was obtained with a size of 2783 bp. Its open reading frame encoded a hypothetical protein of 93.1 kDa. Database search with the program FASTA revealed the highest similarities to the entries At2g21410 (77%), At4g39080 (76 %) and At2g28520 (60 %) from the *A. thaliana *genomic database and also significant similarity on the amino acid level to the genes Stv1 (34 %) and Vph1 (38 %) from *S. cerevisiae*. These two latter genes are coding for isoforms of the yeast 100 kDa subunit whereas all other subunits of the yeast vacuolar ATPase are encoded by one gene each. The sequence alignment between the newly cloned amino acid sequence from *M. crystallinum*, the three isogenes from *A. thaliana *and the two isogenes from yeast (Fig. 1) indicates a structure with two distinct segments distinguished by their degree of similarity: the N-terminus with a low degree of sequence conservation (amino acid 1 to 400) and the C-terminus (amino acid 401 to 816) with a higher degree of conservation between the sequences. This corresponds to the above mentioned domain structure of *S. cerevisiae *VHA-a with a cytoplasmic and a membrane-integrated domain. The heterogeneity in the N-terminus is characterised through a high number of deletions and insertions most remarkably between the amino acid positions Gly84 to Ile85, and Gly141 to Gln142. In contrast to all other VHA-sequences, in yeast-Stv1 this region contains additional sequence insertions of approximately 20 amino acids. Sequence variation between VHA-a from distinct species and paralogues within same species continues until Glu193. Other domains of the N-terminus vary to a lesser extent. The heterogeneity decreases in direction to the membrane-spanning C-terminus.

The N-terminal amino acids of VHA-a that are conserved in all species are characterised by an above average portion of amino acids with an acid or aromatic character. A comparison of the first 400 amino acids of VHA-a from 10 different species showed the presence of 34 amino acids conserved throughout all species, 10 of which have acidic and 8 aromatic residues. In all sequences compared the representation of aromatic amino acids is higher than the average. In yeast it was shown that sequence motifs with aromatic amino acid residues are involved in the targeting of proteins [18]. An assignment of VHA-a sequences from *A. thaliana *and *M. crystallinum *to the distinct yeast isoforms Vph1 and Stv1, in order to define orthologous genes, was not possible on basis of the amino acid sequence alignment. The comparison of the C-termini revealed a higher degree of sequence conservation interrupted through various short insertions or deletions. The sequence was analysed for secondary structures (Predict Protein, EMBL, Heidelberg) and membrane topology (THHM, Denmark). The prediction correlated regions of high sequence conservation with the localisation of putative transmembrane domains (Fig. 1, marked with +). Furthermore, these hypothetical transmembrane domains are in accordance with the membrane-topology suggested by Leng et al. [19] for the yeast Vph1 protein. Mutations in single amino acids of Vph1 have previously allowed the identification of 5 charged amino acids in the membrane spanning helices of the C-terminus whose mutation strongly affected (Lys734, His743, Glu789 and Arg799) or fully inhibited (Arg735) transmembrane H^+^-transport, although they did not affect the assembly of the vacuolar ATPase [19-23]. These functional amino acids are also conserved in Mc-VHA-a.

### Detection of VHA-a in membrane preparations in vitro

**Figure 2:**
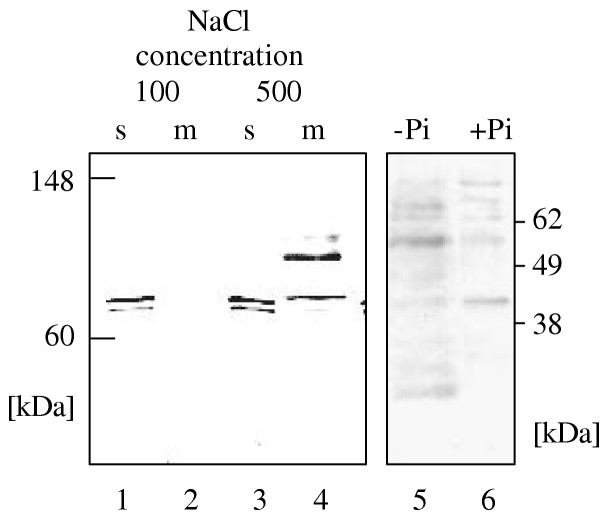
**Immunodetection of VHA-a in membrane and soluble fractions of *M. crystallinum *leaves. **Membranes were isolated in the presence of 100 (1, 2), 500 mM NaCl (3, 4), and without (5) or with (6) protease inhibitor complete^®^, respectively. Membranes and soluble fraction were separated rapidly for (1)–(4) whereas the standard membrane isolation procedure with ultracentrifugation was undertaken for (5) and (6). The 95 kDa band is seen as the dominant protein in the membrane fraction isolated at high salt and to some part in the isolate obtained in the presence of protease inhibitor.

For further examination of McVHA-a, antisera were raised against two polypeptide fragments, i.e. a N-terminal 42 kDa polypeptide and a 13 kDa domain located between predicted transmembrane helices 3 and 4. Both were expressed heterologously in *E. coli *using the primer combination a-nterm-f and a-nterm-r, and a-memb-f and a-memb-r, respectively. The derived PCR-products were cloned into the expression vector pCRT7-NT (Invitrogen, Holland), introduced and expressed in *E. coli*. The 42 kDa and 13 kDa polypeptides were purified to homogeneity by chromatography on Ni-NTA and by preparative SDS-PAGE. The 42 kDa polypeptide was recognized by a purified antibody against yeast Vph1 [13] (not shown). The antiserum produced in guinea pig reacted with the heterologously expressed protein a_N-term _(not shown). The presence of VHA-a was investigated in plant endomembranes and soluble fractions rapidly prepared from 5 week old *M. crystallinum *plants. In the soluble and the membrane-fraction, the serum against McVHA-a_N-term _identified two polypeptides with apparent molecular masses between 65 and 70 kDa (Fig. 2). After increasing the NaCl concentration to 500 mM, the apparently full size 95 kDa band was detected in the membrane fraction corresponding to the expected size of untruncated McVHA-a (Fig. 2). To test whether the addition of NaCl to the membrane preparation affected the purification efficiency of other V-ATPase subunits from the V1-sector, membranes and soluble fractions purified with buffer containing either 100 or 500 mM NaCl were also reacted with antibodies against VHA-E [24] and anti VHA-D_i _recognizing VHA-B and D_i _[25]. The latter authors demonstrated that this antibody marked both VHA-A and -B. The increased NaCl concentration had no effect on the labelling strength of VHA-E and VHA-A/B (not shown). Interestingly, also McVHA-A, -B and -E were detected both in the membrane and soluble fractions.

**Figure 3:**
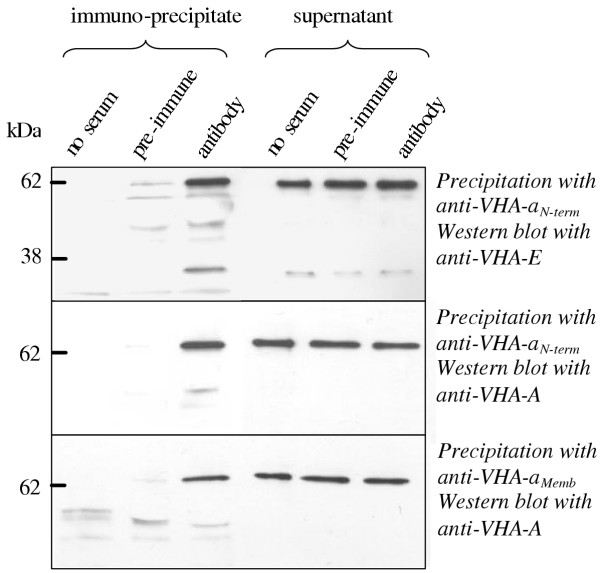
**Co-immunoprecipitation of VHA-E and VHA-A with VHA-a. **Tonoplast enriched membranes of *M. crystallinum *were solubilized in buffer supplemented with 2% (v/v) Triton X-100. Antibody directed against VHA-a, either the N-terminal or membrane part, was added and immunoprecipitation was performed. The pellet samples and part of the supernatant (10–15% of total) were loaded on a SDS-gel, and Western blot was performed with anti VHA-E or A. The band intensities related to loaded sample size indicate that only a fraction of total V-ATPase was immuno-precipitated with the anti-VHA-a antibodies. As controls, immunoprecipitation was performed without added serum and with preimmune serum. With anti-E, monomeric and dimeric band of 30 and 60 kDa was detected by Western blotting in the precipitate obtained with anti VHA-a_N-term_, with anti VHA-A, a 65 kDa band was labelled in separations of both, the precipitates obtained with anti VHA-a_N-term _and anti-VHA-a_Memb_, respectively.

To examine the sensitivity of VHA-a against proteases, membranes were isolated in second approach, in the absence and presence of a protease inhibitor cocktail (complete^®^, Roche, Mannheim, Germany). Without protection from proteolysis, a band at about 50 kDa and a doublet band above 60 kDa were predominant. In the sample with protease inhibitor, the 95 kDa band appeared although as one of five bands of similar intensity if decreasing transfer efficiency of high molecular mass polypeptides from the gel to the membrane is assumed. Apparently, VHA-a is prone to degradation. However, the results also indicate that VHA-a is part of V-ATPase. To further prove that tentative conclusion, immunoprecipitation was performed using anti VHA-a_N-term_and anti VHA-a_memb _followed by Western blot analysis with anti VHA-E and anti-VHA-A (Fig. 3). Each control, i.e. immunoprecipitation without serum and with preimmune serum, gave the expected results of no response (Lanes 1 and 2 in Fig. 3A,3B,3C). With serum, the 55 kDa band of VHA-A and the dimer of VHA-E was seen. Apparently, immunoprecipitation with antibody directed against the N-terminus as well as the membrane part of VHA-a pulled down a complex also containing subunits of the V_1 _section, and thus probably the holocomplex.

### Immunochemical analysis of VHA-distribution

**Figure 4:**
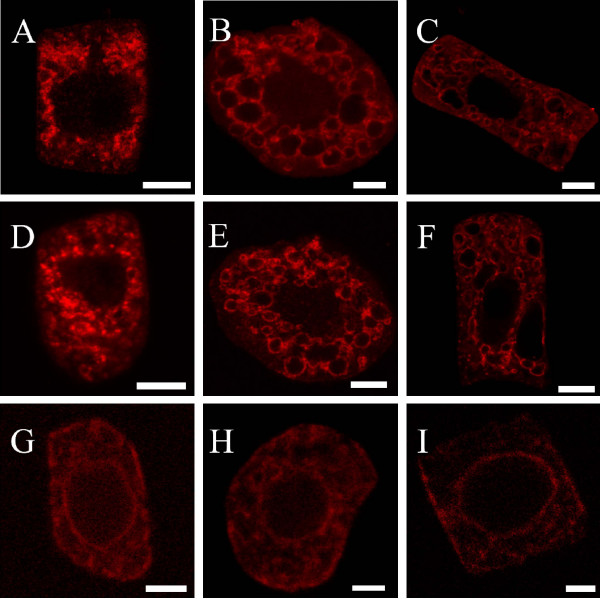
**Localization of VHA-subunits in plant cells, using anti-VHA-A, anti-VHA-E and anti-VHA-a_Nterm_-antibody **Confocal images represent single images of isolated maize root cells. Immuno-staining was performed on maize root cells. Secondary fluorescently labelled antibodies used were anti-rabbit-FITC (A-F) or anti-guineapig (Cy5). Images were colour coded with Adobe Photoshop. Scalebars are 10 μm. Labelling of tonoplast membranes with anti VHA-A (A to C) and anti VHA-E (D-F) in root cells of increasing age, i.e. non-vacuolized to vacuolized (left to right). Anti-VHA-a_N-term _labelling of root cells of increasing age (G-I). Note that the staining pattern of VHA-a_N-term _is distinct from the tonoplast labelling with VHA-A and VHA-E in all cases.

A more detailed analysis of the distribution of the VHA-subunits in the plant cell was performed by immuno-labelling of maize root tip cells. To highlight the distribution of the different VHA-subunits in the cell, squashed maize root cells were incubated with anti VHA-A, anti VHA-E or anti VHA-a_N-term_. Fig 4 shows the staining patterns of the antibodies in young cells devoid of large vacuoles (Fig. 4A,4D,4G), cells with beginning vacuolization (Fig. 4B,4E,4H) and older cells with many vacuoles of 2–5 μm diameter (Fig. 4C,4F,4I). In cells devoid of large vacuoles, anti VHA-E as well as anti-VHA-A marked punctuated compartments whereas in cells with developed vacuoles a nearly complete staining of the tonoplast and an unsteady staining of other small cellular compartments was observed. In a converse manner, anti-VHA-a_N-term _did not stain the tonoplast of cells of various vacuolisation state. The staining with anti-VHA-a_N-term _revealed a distinct reticulate pattern and a staining of the nuclear membrane reminding of ER-labelling.

**Figure 5:**
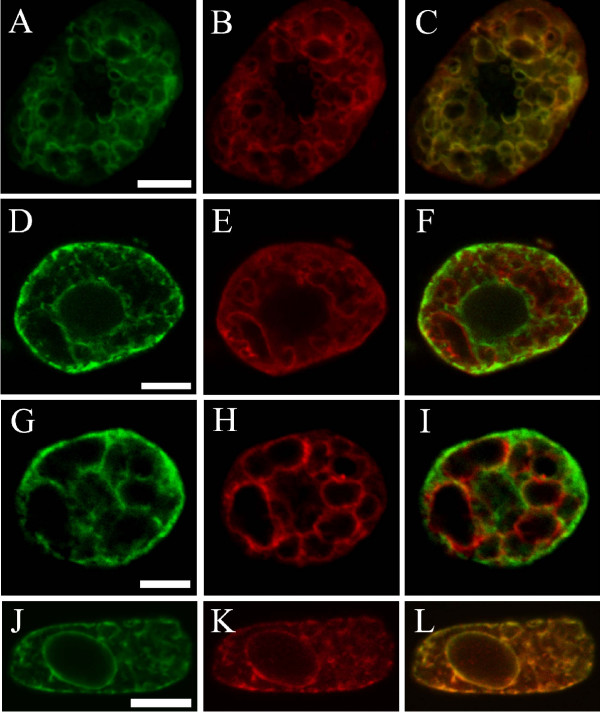
**Co-localization of VHA-subunits with the tonoplast marker γ-Tip and calreticulin, a marker for the ER. **Confocal images represent single images of isolated maize root cells which were immuno-probed with antibodies directed against marker polypeptides of the vacuolar membrane (γ-Tip) and endoplasmatic reticulum (calreticulin) and simultaneously treated with anti-VHA-antibodies. Secondary fluorescently labelled antibodies used were anti-rabbit-FITC (A), anti rabbit-Cy3 (B, E, H), anti-mouse-FITC (D, G, J) or anti-guineapig-Cy5 (K). Images were colour coded with Adobe Photoshop. Scalebars are 10 μm. In each row, the immuno-decoration with the marker, with the VHA-subunit specific antibody and the superposition of both is shown. (A-C) Root cell labelled with γ-Tip (A) and VHA-A (B). Note the complete co-localisation of the both markers on the tonoplast of small vacuoles (C). (D-F) Double-staining with calreticulin (D) and VHA-A. (G-I) Double-labelling of a root cell with calreticulin (G) and VHA-E (H) reveals a similar result as with VHA-A. Tonoplast labelling and ER-staining are distinct. (J-L) Co-labelling with calreticulin (J) and VHA-a_N-term _reveals a complete co-localisation of the two signals on the ER.

A double immuno-labelling-technique [26] was then employed to identify the antibody-marked intracellular compartments in detail. The selected antibodies were directed against the aquaporin γ-Tip located in the tonoplast of the lytic vacuole (anti γ-Tip; [27]) and against marker components of the endoplasmic reticulum (ER) (anti-calreticulin). The results of this immuno-staining are shown in Fig. 5. In cells with developed vacuoles, anti-γ-Tip-labelling of the tonoplast (Fig. 5A) co-localised completely with anti-VHA-A (Fig. 5B,5C). Anti VHA-E labelled similar structures as anti-VHA-A and co-localised also with anti-γ-Tip on the tonoplast (not shown). A co-staining of root cells with anti-VHA-A and the ER-marker anti-calreticulin was then performed. Anti-calreticulin marks a specific ER-network including the nuclear membrane (Fig. 5D,5G,5J). The double labelling with antibodies against VHA-A (Fig. 5E) or VHA-E (Fig. 5H) showed no significant co-localisation of the typical tonoplast staining with the ER-marker. When performing a co-labelling of maize root cells with anti-calreticulin (Fig. 5J) and anti-VHA-a_N-term _(Fig. 5K) revealed a complete co-labelling of the two markers (Fig. 5L).

**Figure 6:**
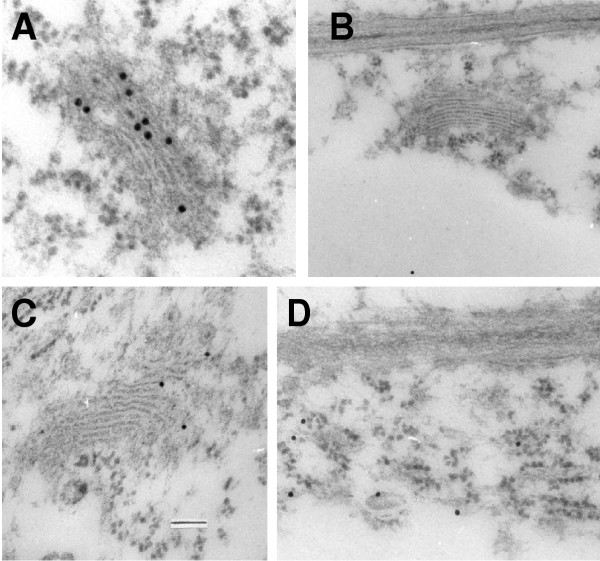
**Immunogold-based localisation of VHA-a. **Ultra-thin cross sections of maize root cells in 1 mm distance to the tip were decorated anti VHA- (A), premmune serum, (B), anti VHA-a_N-term_-antibody  (C,D), respectively. Sections were washed, treated with secondary antibody linked to 15 nm gold particles and visualized in an electron microscope.

For high resolution, immunogold labelling with anti VHA-a_N-term _and anti VHA-A was performed on ultra-thin cross sections of maize root cells (Fig. 6). With anti VHA-a pronounced label with gold particles was detected in ER membranes (Fig. 6D), and occasionally a weak labelling of the Golgi apparatus (Fig. 6C). A labelling of tonoplast membranes was not found, indicating that VHA-a_N-term _is predominantly located on the ER. For comparison, immuno-gold analysis of ultrathin cross sections with anti-VHA-A. revealed labelling of the tonoplast (not shown) and a labelling of the Golgi apparatus significantly stronger than with anti-VHA-a (Fig. 6A).

### Fluorescence resonance energy transfer between VHA-subunits in vivo

**Figure 7:**
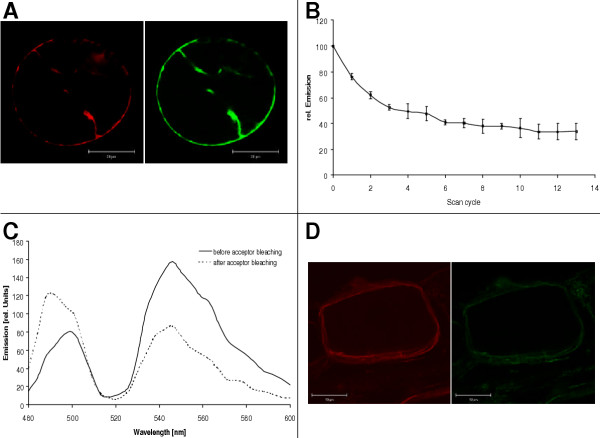
**FRET between VHA-subunits co-expressed in protoplasts of *A. thaliana *and onion epidermis cells**. (A) Mesophyll protoplasts of *A. thaliana *were simultaneously transformed with p35S::VHA-a/YFP and p35S::VHA-c/CFP using the polyethylene glycol method. After 20 h, fluorescence emission from protoplasts was measured following excitation at 458 nm and 514 nm, and image analysis in the range of 470 – 500 nm for CFP and 560 – 585 nm for YFP fluorescence, respectively. (B) Acceptor bleaching in dependence on scan numbers. For this experiment, protoplasts expressing VHA-a-YFP were excited at 514 nm and emission was recorded between 550 and 600 nm. (C) Emission spectra of VHA-c-CFP and VHA-a-YFP before (solid line) and after (broken line) acceptor bleaching. (D) FRET between VHA-A-YFP and VHA-B-CFP after co-expression in onion epidermis cells. Excitation was achieved at 458 nm, and 2D emission images were recorded in the range of 470 to 510 nm (CFP), and 550 to 600 nm (YFP).

FRET allows to investigate protein-protein interaction *in vitro *and *in vivo*. Both partners have to carry fluorescent labels with overlapping emission (donor fluorophore) and excitation spectra (acceptor) and need to be situated in close proximity. Half maximum energy transfer takes place at distance of the Förster radius R_0_. Cyan and yellow fluorescent proteins constitute such a FRET pair and were fused to the C-termini of various subunits of V-ATPase. Under the assumption of freely rotating fluorophores, R_0 _is close to 5 nm. The size of the V-ATPase complex is about 15 nm (diameter) × 25 nm (length from lumen side to tip of head). *Arabidopsis *protoplasts were co-transformed with vector constructs of VHA-a fused to YFP and VHA-c fused to CFP under the control of the 35S promotor. Upon excitation of doubly labelled protoplasts at 458 nm both, CFP and YFP showed strong fluorescence (Fig. 7A). Fluorescence emission spectra were recorded using a double dichroic mirror which exhibits high reflectivity at ~514 nm. Therefore, the two emission maxima were separated by a minimum (Fig. 7C). This fact renders the quantitative analysis of the FRET efficiency due to the decrease in donor and increase in acceptor fluorescence more difficult. Alternatively, the method of acceptor bleaching can be used to verify energy transfer [28] and can be seen in Fig. 7B, the fluorescence intensity of YFP decreased rapidly within 10 scan cycles upon excitation at 514 nm due to photobleaching to a residual intensity attributable to auto-fluorescence. Simultaneously, the donor fluorescence increased substantially, thus providing direct evidence for energy transfer from CFP to YFP. From the increase in CFP fluorescence upon acceptor bleaching an overall FRET efficiency of ~0.45 is estimated. Assuming freely rotating fluorophores (K^2 ^= 2/3) this corresponds to a distance between VHA-a, and VHA-c of ~5.4 to 7.2 nm (mean 6.3 ± 0.8 nm). Here it has to be pointed out that each ring of the rotor V_0 _contains 5 VHA-c-subunits and one VHA-c"-subunit. Hence, dependent on the position of the VHA-c/CFP-subunits in the ring different distances between CFP-labelled VHA-c subunits and the YFP-labelled VHA-a subunit will result.

Similar experiments were performed with onion epidermis cells (Fig. 7D) co-transformed with VHA-A/YFP and VHA-B/CFP, and VHA-B/CFP and VHA-H/YFP, respectively. This system was employed for two reasons, (i) to confirm and extent the results from protoplasts and (ii) to work in turgescent cells, not previously subjected to a protoplast isolation stress. Emission spectra were recorded before and after photobleaching of acceptor. Decreases in acceptor and increases in donor fluorescence intensities, respectively, were smaller for these pairs of VHA-fusions than for VHA-a-YFP and VHA-c-CFP (shown in Fig. 7C). Nevertheless there was significant FRET in the case of VHA-A/YFP and VHA-B/CFP and some indication of FRET in the case of VHA-B/CFP and VHA-H/YFP, whereas the co-transformed pair of VHA-A/CFP and VHA-H/YFP gave no FRET. It should be noted that in addition to the highly expressed fusion proteins, untagged endogenous subunits still are present in the cell. Under such condition, formation of partial subcomplexes with possibly varied FRET properties may not be ruled out.

## Discussion

**Figure 8:**
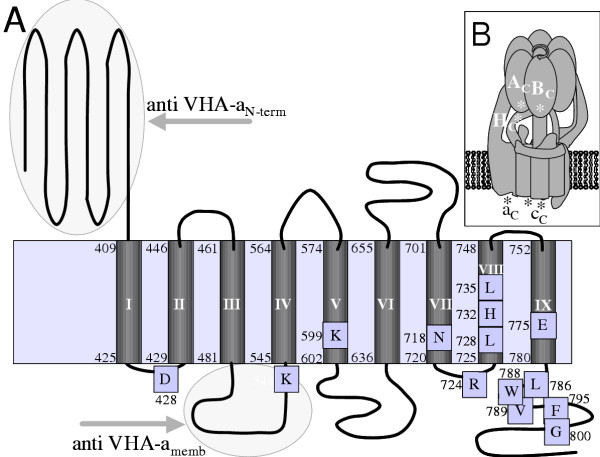
**Membrane topology of plant VHA-a and location of the C-termini of individual subunits based on the FRET data. **Based on amino acid sequence analysis and similarity with Vph1 (Leng et al. 1999), the topology of Mc-VHA-a is depicted in (A). The relative location of amino acid residues essential for proton pumping or structure are indicated with boxes. The numbers indicate the amino acid positions. In (B), the results from the FRET experiments are summarized: Asterisks tentatively mark the positions of the C-termini where the GFP variants have been fused to.

The set of subunits that assemble plant V-ATPase has recently been completed by similarity searches in plant genomic and EST sequences using information on yeast VMA and other orthologues [5,6]. The presence of a subunit of about 100 kDa in the functionally active plant vacuolar ATPase has been under discussion for a long time [14]. Here, for the first time, a cDNA coding for a plant VHA-a was cloned and characterised. McVHA-a as well as the homologous *Arabidopsis *gene products contain all charged amino acids that have been shown to be essential for proton-translocation at conserved positions in the membrane spanning region of the C-terminus (Fig. 8). Additional evidence for an essential function of VHA-a in V-ATPase was first obtained for V-ATPase of *Bos bovis*. The specific V-ATPase-inhibitor bafilomycin [29] was shown to bind to the 100 kDa VHA-a subunit and not, as previously suggested to the proteolipid VHA-c. The results were confirmed for all tested species [30]. Bafilomycin also is a potent inhibitor of plant V-ATPase and is routinely used to distinguish V-ATPase-dependent ATP hydrolysis from background activity [31]. In 1999, Li and Sze [14] detected two unknown polypeptides with apparent molecular masses of 63 and 54 kDa in purified catalytically active V-ATPase but no polypeptide with a molecular mass of about 100 kDa. The authors hypothesized that plant V-ATPase is active in the absence of the 100 kDa subunit. Another explanation for this observation could be the sensitivity of VHA-a to degradation through proteases [32], and the detected unknown polypeptides could result from limited proteolysis of VHA-a, producing subunit-fragments still capable of transporting protons. The immunoblots with soluble fractions and membrane-preparations of *M. crystallinum *(Fig. 2) incubated with anti VHA-a_N-term _support this hypothesis. The 65 kDa fragment in the cytoplasmic fraction is likely to derive from a proteolytic processing, releasing the soluble portion of McVHA-a. Accordingly, high NaCl concentration in the purification medium could inhibit involved proteases. The hypthesis is supported by the experiment with protease inhibitor cocktail, where the 95 kDa subunit was detected as band with intermediate intensity, although only among other bands that were immuno-responsive to anti-VHA-a antibody. Thus even the protease inhibitors could not fully suppress proteolysis. Immunoprecipitation with the antibodies anti VHA-a_N-term _and anti VHA-a_memb _further proved that both the N-terminal hyrophilic portion and the membrane sector of VHA-a are associated with a complete V-ATPase complex.

In plants, immuno-cytochemical examinations of the subcellular localisation of single subunits or holocomplexes (mostly using antibodies directed against VHA-A) have previously indicated a distribution of V-ATPase among nearly all endomembranes of the secretory pathway including the plasmalemma [1-3], [33-35]. These findings were supported by measurement of bafilomycin-sensitive ATPase activity in selectively purified endomembranes of plants [36-38].

In our examination the antisera against VHA-A and -E showed a staining of the tonoplast and to a lesser extent also of the ER and Golgi-Apparatus (data not shown). VHA-A and E are part of the cytoplasmically exposed V_1_-structure of V-ATPase. The presence of both subunits on all these membranes shows the presence of fully assembled V-ATPase.

The presence of the active V-ATPase in plants is not only necessary at the tonoplast, since Matsuoka et al. [38] could show that the activity of the vacuolar ATPase on the ER and the GA is necessary for correct targeting of soluble storage proteins to the vacuole. The presence of active V-ATPase on the prevacuolar compartment was concluded from the acid pH-optimum of enzymes involved in vacuolar transport, for example the activity of the vacuolar sorting receptor BP-80 whose action is strictly pH dependent [21]. For this reason a similar staining pattern of all used anti-VHA antisera would have been anticipated, but our results showed distinct staining patterns of anti-VHA-A and -E on the one hand and anti-VHA-a_N-term _on the other hand. Immuno-labelling indicates that VHA-a_N-term _antiserum exclusively labels the ER with some rare association on Golgi stacks. These results are surprising since the sequence features of McVHA-a suggest an essential involvement of VHA-a in proton transport. A possible explanation might be the presence of three different isoforms in *A. thaliana *and *O. sativa *which all share the localisation of the charged amino acids responsible for proton-translocation (Fig. 1). Based on our results, we suggest that the isoform of VHA-a recognised by anti VHA a-_Nterm _(an antibody which was generated against the isoform-specific N-terminus) is exclusively associated with V-ATPase localised on the ER. The hypothesis of a compartment-specific localisation of VHA-a subunits is supported through several findings in plants and yeast. In yeast all known subunits and chaperones of the V-ATPase are encoded by one gene. Only the 100 kDa VHA-a is encoded by two different isoforms (Vph1 and Stv1) [13]. In general the subunit-isogenes of the V-ATPase have a very high degree of similarity within each species [16,39]. In a converse manner, the sequences of the VHA-a isogenes are very heterogenic in *S. cerevisiae *and *A. thaliana *(Fig. 1) especially in the cytoplasmic region of the N-terminus [40]. A differential localisation was shown for the two yeast isoforms (Vph1 and Stv1) [12]. A detailed examination through Kawasaki-Nishi et al. [40] showed a localisation of Vph1 on the tonoplast, while Stv1 was detected on the late Golgi-apparatus and the prevacuolar compartment of yeast. By expressing chimeric proteins composed of the N-terminus of Stv1 and the C-terminus of Vph1, and vice versa, the authors were able to show that the N-terminus defines subcellular sorting. Following selective enrichment of the differentially localised complexes, both types of V-ATPases were shown to differ in their stability of the V_0_/V_1_-complex and in their coupling efficiency [41]. The N-terminus was responsible for the differential coupling activity, whereas the C-terminus mediated the differential dissociation.

In plants, Matsuoka et al. [38] were able to distinguish between two different V-ATPase activities through their differential response to the V-ATPase inhibitor bafilomycin. These V-ATPase activities were localised in distinct membrane fractions of the secretory pathway including the vacuolar compartments. An antibody directed against the V-ATPase holoenzyme revealed significant differences in immuno-staining of endomembrane and vacuolar fractions. These and our results on sequence properties of the different plant VHA-a isoforms and the intracellular localisation of VHA-a support the hypothesis that at least two different V-ATPase activities exist in plants, differing in intracellular localisation and sensitivity to bafilomycin. The target of bafilomycin is VHA-a [29]. Thus, both types of V-ATPases might be distinguished through the presence of different isoforms of VHA-a.

Our results from FRET analysis also allows to make some structural assignments: From crystal structure of F-ATP synthase, the C-termini of subunit β, homologous to VHA-A, and subunit α, homologous to VHA-B, are located in close vicinity and oriented to the membrane [42]. Occurrence of FRET between VHA-A/YFP and VHA-B/CFP supports the same structural arrangement in V-ATPase. Following crystalization of isolated yeast VHA-H [16], the structure was fitted in 3D reconstructions of plant V-ATPase based on electron microscopic analysis [43] and suggests localization of the C-terminus of VHA-H to the head structure in proximity to VHA-B. *In vivo*-FRET in protoplasts expressing VHA-B/CFP and VHA-H-YFP confirms the orientation of the C-termini of VHA-B and H in close vicinity. It should be noted that co-expression of other pairs of chimeric donors and acceptors such as VHA-E/CFP and VHA-c/YFP did not elicit FRET after excitation of CFP (not shown). The assumed location of the C-terminus of VHA-c in the lumen of the endomembrane compartments and the C-terminus of VHA-E most likely in the vicinity of the head is in agreement with the negative result, i.e. the absence of FRET between VHA-E/CFP and VHA-c/YFP. The studies exemplify the suitability of FRET to analyse structural features of V-ATPase *in vivo*. The efficient but variable FRET in cells expressing VHA-c/CFP and VHA-a/YFP allows two conclusions: First, the C-termini of VHA-c and VHA-a are likely to be located on the same, i.e. luminal, side of the endomembrane compartments supporting the topological model with nine transmembrane-domains of VHA-a in plants as previously suggested for yeast [22,23] (Fig. 8). Second, a significant portion of total VHA-a is located in the neighbourhood of VHA-c. The calculated distance of 5.4 to 7.2 nm between donor and acceptor fluorophore corresponds to the diameter of the proteolipid-ring of the rotor, consisting of 5 subunits of VHA-c and 1 VHA-c", respectively. The results confirm that VHA-a is part of the functional complex and not only involved in V-ATPase assembly. More than half (cf. Fig. 2), and possibly all V-ATPase complexes contain the holopolypeptide of 95 kDa.

## Conclusions

The analysis of the primary structure of plant VHA-a revealed the presence of amino acid residues that are essential for proton pumping in yeast. Employing immuno-co-precipitation and FRET it could be demonstrated that subunits VHA-a and VHA-H are part of the V-ATPase complex of plants. Furtheron it is shown that one distinct VHA-a subunit isoform is localized on the ER. The study also shows the usefulness of FRET to study multisubunit protein structures in vivo and in vitro.

## Methods

### Plant growth

*Mesembryanthemum crystallinum *and *Arabidopsis thaliana *were grown in hydroponics and soil culture, respectively, as described in [10,44]. Growth conditions were 120 μmol quanta m^-2 ^s^-1^, 60% relative humidity, 20°C, and a daily photoperiod of 12 h duration. Rosette leaves from 3- to 5-week-old *Arabidopsis *plants were taken for protoplast transfection. *Zea mays *and *Hordeum vulgare *were germinated on filter paper in the dark at 25°C for 48 h. Cells were isolated from the first 2 mm of the growing root tip. Onion epidermis was stripped from onion bulbs obtained from a local market.

### Membrane isolation

Leaves (50 g) of *M. crystallinum *were homogenized in a buffer containing 250 mM sucrose, 50 mM Tris-Cl, pH 8.0, 4 mM ethylenediamine tetraacetic acid (EDTA), 4 mM dithiothreitol and a few crystals of phenylmethylsulfonylfluoride [45]. As indicated in a set of experiments, either NaCl was added at 100 or 500 mM concentration or complete protease inhibitor^® ^cocktail (Roche, Mannheim, Germany) was added throughout the procedure. Following differential sedimentation and gradient centrifugation, tonoplast enriched membranes were recovered from a 30%/35% sucrose interphase, sedimented, frozen in liquid nitrogen and stored at -80°C.

### Gel electrophoresis and Western blot detection

Membrane proteins were separated on 12.5% sodium dodecylsulfate polyacrylamide gels, transferred to nitrocellulose and probed with anti-VHA-E [46], anti-VHA-A (kind gift of Dr. R. Ratajczak and Prof. U. Lüttge, TU Darmstadt, Germany) raised in rabbit or anti-VHA-a raised in guinea pig. Following incubation with primary and secondary antibody conjugated with peroxidase, detection was achieved with the lumilight^® ^system according to the supplier (Roche, Mannheim, Germany).

### Immunoprecipitation

For immunoprecipitation, membranes were solubilised in 50 mM Tris-Cl, pH 7.5, 150 mM NaCl, 1 mM EDTA and 2% (v/v) Triton X-100, 5 μl anti VHA-a antiserum was added, and the samples were shaken at room temperature for 45 min. Then 150 μl protein A-sepharose equilibrated in the same buffer was added. After 15 min, the suspension was placed on a cushion of 1 ml of 40% sucrose and spun at 10,000 × g for 1 min. The sediment was washed thrice with 50 mM Tris-Cl, pH 7.5, 150 mM NaCl, 1 mM EDTA, 1% (v/v) Triton X-100 and 0.1 % (w/v) SDS, and finally once in 125 mM Tris-Cl, pH 6.8. The sediment was boiled in loading buffer and analysed by Western blot using rabbit antisera raised against VHA-E or A.

### Anti-VHA- a antibody preparation and other antibodies used in this study

Two antibodies against specific domains of VHA-a were raised in rabbits, and denominated anti-VHA-a_N-term _and anti-VHA-a_Memb_. For both the corresponding cDNA fragments of Mc-VHA-a were amplified by PCR using primer combinations a-nterm-f (ATG CGA TCG GAG CCG ATG CAA) and a-nterm-r (TTC ACC CAA CTC ATC GGT GG) encoding the 42 N-terminally located fragment, and a-memb-f (CTT CCA AAG CCC TTT ATT ATG) and a-memb-r (TCA CTC ATG TCC ACC ATG TCA ATC) encoding the polypeptide loop of about 13 kDa located between transmembrane domain 3 and 4 according to the topological model of Vph1p of *S. cerevisiae *[47]. The gene fragments were cloned into the vector pCR-T7-NT-Topo (Invitrogen, The Netherlands) and transformed into *E. coli *JM109. The 6x-his-tagged proteins were expressed, purified by chromatography on Ni-nitrilotriacetate columns, separated by preparative SDS-PAGE, excised as protein bands, eluted and used for immunization (Pineda, Berlin). In addition, antisera against subunits VHA-A (kind gift of Dr. R. Ratajczak and Prof. U. Lüttge, TU Darmstadt, Germany), VHA-E, calreticulin ([48], kindly provided by Andrew Smith, Oxford, UK), Jim 84 ([49] kindly provided by Chris Hawes, Oxford, UK), γ-TIP ([27]; kindly provided by John C Rogers, Washington State University, USA) were used in the co-localization studies.

### Construction of fusions between VHA subunits and variants of green fluorescence protein (GFP)

Mc-VHA-a and -c were cloned into the vectors pECFP/pEYFP (Clontech, Palo Alto, USA) in a site-directed manner after amplification from cDNA [10] using the primers a-ges-BamHI-f (AAA AGG ATC CAT GCG ATC GGA GCC GAT GCA A) and a-ges-NcoI-r (AAA AAC ATG GCC TCT TCT TCT TCA CCA ATC GT), McVHA-c with c-ges-BamHI-f (AAA AGG ATC CAT GTC AAC CGT CTT CAA TGG) and c-ges-NcoI-r (AAA ACC ATG GCT GCC CTT GAC TGT CCA GCT CG). Mc-VHA-A and Mc-VHA-H were cloned as described in [10]. The constructs were introduced into the vector p35SGFP [50], so that the chimeric genes were placed under control of the 35S promoter and the original GFP gene was lost. The same strategy was used to produce Mc-VHA-A, -B and -H gene fusions with variants of GFP.

### Protoplast isolation and transformation methods

Protoplasts were gently sedimented by centrifugation, resuspended in W5 medium, sedimented again, resuspended in MMG medium (0.4 M mannitol, 15 mM MgCI_2_, 4 mM morpholinoethane sulfonic acid, KOH , phl 5.7) and checked for sufficient intactness in the microscope. In short, 1 mm leaf slices of 3- to 5-week-old *Arabidopsis *plants were vacuum-infiltrated and cell walls were digested in media containing 1.5 % (w/v) cellulase R10 and 0.4 % (w/v) macerozyme R10. Protoplasts were gently sedimented by centrifugation, resuspended in W5 medium, sedimented again, resuspended in MMG medium (0.4 M mannitol, 15 mM MgCl_2_, 4 mM morpholinoethane sulfonic acid, KOH, pH 5.7) and checked for sufficient intactness in the microscope. 110 μl PEG-medium (4 % (w/v) polyethylene glycol 4000, 0.2 M mannitol, 0.1 M CaCl_2_) and 20 μl plasmid DNA (3 μg/μl) were added to 100 μl protoplast suspension. The samples were incubated at room temperature for 15 min and then consecutively diluted with 0.5, 1, 2 and 4 ml W5-medium with 15 min incubation steps in between (154 mM NaCl, 125 mM CaCl_2_, 5 mM KCl, 2 mM morpholinoethane sulfonic acid, KOH, pH 5.7). Following 24 h incubation at 25°C, sedimented protoplasts were used for analysis.

Cells of onion epidermis were placed on filter paper soaked with one-strength MS basal medium in petri dishes and were transiently transformed with a biolistic approach. Gold particles (1.6 μm, 60 mg/ml) were suspended in 50 % glycerol. 8.33 μl of the suspension were mixed with 8.33 μl plasmid DNA (1 μg/ μl), 8.33 μl 2.5 M CaCl_2_, 3.33 μl 0.1 M spermidine. Sedimented gold particles were consecutively washed with 70 % and 100 % ethanol and resuspended in 8 μl 100 % ethanol, loaded on a macro carrier for transformation with the Particle Delivery System using a rupture disc of 1100 psi (PDS-1000/He, Biorad, Hercules, USA). The distance between macrocarrier and tissue was 12 cm. The epidermis tissue was incubated for about 20 h at room temperature in the dark prior to analysis.

### Immuno-fluorescence labelling and image acquisition by confocal laser scanning microscopy (CLSM)

Immuno-labelling was performed according to [26]. In brief, cells were fixed in 3.7 % para-formaldehyde (10 mM MgSO_4_, 10 mM EGTA, 1 × phosphate buffered saline, pH 6.8), washed, permeabilised in 0.5% Triton X-100 and washed again. Following blocking of non-specific binding sites with 1 % bovine serum albumin, primary antibody was added for over night at 4°C. Washed samples were incubated with secondary antibody labelled with Cy3, Cy5 or FITC for 1 h. Double labelling was performed by combined application of primary antibodies from rabbit and guinea pig. Slides were mounted with Citifluor Mounting Medium. Fluorescence analysis was performed with a confocal laser scanning microscope Leica TCS-SP2 (Leica, Heidelberg, Germany) equipped with three lasers and excitation wavelengths of 458, 476, 488, 514, 568 and 633 nm. The double dichroic mirror DD488/543 was used for fluorescein isothiocyanate (FITC), and for Cy5 the triple dichroic mirror TD488/543/633 was used. Background was controlled and photomultiplier voltage (800 V) selected for maximum sensitivity in the linear range.

### Immunogold-labelling and electron microscopy

Cells were fixed in 2.5% glutaraldehyde in EM buffer (50 mM KH_2_PO_4_, 50 mM NaH_2_PO_4_, pH 7.0) for 45 min, washed with EM buffer and dehydrated with a series of increasing concentration of acetone. Samples were embedded in epoxyresin (Transmit EM, TAAB laboratories equipment, Berkshire, Great Britain), cut into ultra-thin cross-sections of 60–70 nm and immobilized on 200 mesh gold nets. Immuno-decoration was performed with antibody diluted in Tris-buffered saline (TBS, 10 mM bovine serum albumin and 0.05 % (w/v) NaN_3_) for an hour. Samples were washed five times and incubated with secondary antibody conjugated to 15 nm gold particles. The samples were stained with 0.1 % (w/v) uranyl acetate for 5 s and afterwards with 2 % lead citrate. The samples were analysed with an electron microscope (H500, Hitachi, Japan) at 75 kV.

### Confocal microscopy of GFP-fusion proteins and FRET-measurement

Transformed protoplasts and onion epidermis cells were examined for the localisation of the CFP/YFP-fused proteins using the same CLSM set-up as mentioned above. Autofluorescence of 10 protoplasts, as well as reference spectra of YFP and CFP-derived fluorescence were recorded in the spectral range of 480 to 700 nm, averaged and used for corrections. Excitation was recorded at 458 nm (CFP and FRET) and 514 nm (YFP), respectively. Scan speed was 800 Hz. Acceptor dye was bleached with 100 % laser intensity. Emission spectra were recorded and averaged from 20 transformed protoplasts. For a first estimate of transfer efficiency, a Foerster radius for green fluorescence protein variants of R_o _= 5 nm [52] was used to calculate the donor/acceptor distance via the equations E = (I_CFP/bleached _- I_CFP/unbleached_)/I_CFP/unbleached _and R = ((R_o_^6^/E)-R_o_^6^)^1/6^, where E is the transfer efficiency, and I_CFP _the fluorescence emission intensity in the CFP peak.

## Abbreviations

CFP: cyan fluorescence protein; CLSM: confocal laser scanning microscope; FRET: (Förster) fluorescence resonance energy transfer; PAGE: polyacrylamide gel electrophoresis; Stv1: VHA-a subunit isogene in yeast; VHA: vacuolar H^+^-ATPase; Vph1: VHA-a subunit isogene in yeast; YFP: yellow fluorescence protein

## Authors' contributions

CK: VHA-a sequence analysis, immuno-cytochemistry, transient expression systems, preparation of anti VHA-a_N-term_; TS: co-transfection of protoplasts and epidermis cells, CLSM analysis; SB: immuno-co-localisation and discussion; SS: co-immuno-precipitation; MH: preparation of anti VHA-a_Memb_; BS-J: immuno-co-localisation and discussion; JR and MS: FRET analysis; DG: transformation and construct design, KJD: project design and supervision.
